# Factors associated with preoperative attrition in bariatric surgery: a protocol for a systematic review

**DOI:** 10.1186/s13643-018-0855-x

**Published:** 2018-11-28

**Authors:** Tamasin Taylor, Ofa Dewes, Nalei Taufa, Wendy Wrapson, Richard Siegert

**Affiliations:** 1 Auckland University of Technology, 90 Akoranga Drive, Northcote, Auckland, 0627 New Zealand; 2 Maurice Wilkins Centre for Molecular Biodiscovery, Auckland, New Zealand; 3 University of Auckland, Auckland, New Zealand; 4 Auckland University of Technology, Auckland, New Zealand

**Keywords:** Preoperative, Bariatric surgery, Obesity, Disparities, Systematic review, Humans, Morbid, Obesity surgery, Attrition

## Abstract

**Background:**

Bariatric surgery results in substantial medical and economic benefits; however, independent studies typically report high patient preoperative attrition rates. Studies have identified individual characteristics and sociodemographic variables of those who complete the surgery compared to those who do not. The aim of the present protocol is to outline a systematic review focussed on identifying the sociodemographic, medical, cultural, psychological, and patient-led factors affecting preoperative attrition in clients who were enrolled in bariatric surgery programmes.

**Methods/design:**

The databases Scopus, CINAHL, PsycINFO, Web of Science, and MEDLINE will be searched for retrospective, prospective, and cross-sectional observational studies that have identified any sociodemographic, medical, cultural, psychological, and patient-led factors affecting preoperative attrition in clients who are enrolled in a bariatric surgery programme. English-language articles published between 1997 to 2020, inclusive of adults 18 years or older, will be included in the review. This protocol has been registered in PROSPERO, registration number; CRD42017068557.

**Discussion:**

Presently, there are studies and reviews investigating population-based utilisation and access to bariatric surgery; however, there is a need to review the reasons behind preoperative bariatric surgery patient attrition once selected for bariatric surgery. The results of the review will highlight potential systematic disparities in patient attrition, where gaps in knowledge remain for further investigation, and suggest areas where countermeasures may be focussed for decreasing attrition rates.

**Systematic review registration:**

PROSPERO CRD42017068557

**Electronic supplementary material:**

The online version of this article (10.1186/s13643-018-0855-x) contains supplementary material, which is available to authorized users.

## Background

Bariatric surgery is now recognised as the most effective procedure to treat patients with obesity and obesity-related co-morbidities as well as resulting in significant short and long-term savings in costs of healthcare resource utilisation [1–5]. The National Institutes of Health (NIH) criteria for bariatric surgery is a BMI > 40 kg/m^2^, or greater, or a BMI > 35 kg/m^2^ or greater with significant obesity-related comorbidities [6]. There are four different bariatric surgery operative procedures presently used worldwide including the gastric bypass (restrictive or malabsorptive), alone or in combination with vertical banded gastroplasty, laparoscopic adjustable gastric banding (restrictive), vertical banded gastroplasty (restrictive), and biliopancreatic diversion, and duodenal switch (primarily malabsorptive) [1].

Bariatric surgery can result in significant and permanent weight loss and the elimination or improvement of most obesity-related comorbidities. These include diabetes, hypertension, hyperlipidaemia, obstructive sleep apnoea, gastroesophageal reflux disease, cardiac dysfunction, osteoarthritis and low back pain, non-alcoholic fatty liver disease, intertriginous dermatitis, stress incontinence, symptoms of depression, and eating disorders [1]. Additionally, bariatric surgery has shown to be cost-effective and pay for itself within 4 years compared with no operative management [1]. In terms of post-operative risks from the operative stage to 5 years, the complication rates associated with surgery range from 10% to 17% and reoperation rates are around 7% [7, 8]. Mortality associated with the surgery is low, ranging between 0.08 and 0.35% [8].

Some complications resulting from bariatric surgery that can occur within 30 days of surgery include wound infection, deep-vein thrombosis, small bowel obstructions, abdominal leaks, and death [9]. Patients who smoke are at an increased risk of poor wound healing, anastomotic ulcers, and overall impaired health [10]. Aside from smoking, other pre-existing patient-risk factors include extremes of BMI, prior venous thromboembolism, mobility limitations, obstructive sleep apnea, age greater than 50, coronary artery disease, pulmonary disease, and male gender [11].

Longitudinal studies show worse post-surgery outcomes for those with less behavioural compliance to preoperative programs. For example, patients who lose the least weight at 24 months post-surgery have been found to show less compliance with pre-surgical requirements including attending few medical appointments and low adherence to behavioural changes (i.e. diet and exercise) [12–14]. A review identified that gradual post-surgical weight increases in patients between 2 and 5 years after having a sleeve gastrectomy surgery were attributed to maladaptive eating and lack of exercise [15]. In comparison, significant weight loss at 5 years was related to lifestyle modification scores that were higher than the scores of patients with insufficient weight loss. Together, the literature indicates that patients who follow preoperative instructions and succeed in making healthier lifestyle changes are more likely to have positive outcomes from surgery.

These results suggest the importance of establishing healthy lifestyle behaviours at the pre-surgical stage. To reduce complications and improve weight loss, many bariatric programs require patients to achieve a preoperative weight loss of approximately 10% of excess body weight above a BMI of 25 to increase fitness and mobility, to quit smoking and show improved glycemic control through diet [8–11]. There are limited understandings of which patients are specifically more likely to adhere to the preoperative requirements, although one review found that patients who received surgery were more likely to be white, female, and have private medical insurance [16]. This suggested that sociodemographic factors may be a contributing factor to preoperative program retention.

In summary, it is important that barriers to preoperative care are synthesised so that providers can better support their patients through the preoperative stage and achieve best possible post-surgical outcomes [13]. This protocol describes a systematic review that aims to identify the sociodemographic, medical, cultural, psychological, and patient-led factors related to preoperative attrition in patients who were enrolled in bariatric surgery programmes.

## Methods

### Context/statement question

The aim of the systematic review is to identify the sociodemographic, medical, cultural, psychological, and patient-led factors affecting preoperative attrition in bariatric surgery clients who were enrolled in a bariatric surgery programme. The proposed systematic review will therefore answer the following question:

What are the factors identified in the existing literature as affecting preoperative bariatric surgery attrition in clients who are eligible for bariatric surgery compared to patients who do go through with surgery?

Objective 1: To synthesise the rates and reasons for attrition for bariatric surgery clients in the preoperative period in the form of a systematic review.

Objective 2: To include in the review any grouping factors (eg., sociodemographic or medical factors) that may be attributed to attrition in the bariatric surgery preoperative period compared with patients who do go through with surgery. The PRISMA-P checklist [17] guided the writing of this protocol (see Additional file 1). The PRISMA guidance [18] will be used to guide the systematic review manuscript.

### Eligibility

#### Participants/population

The population of interest are clients aged 18+ who were enrolled into a private or publically funded bariatric surgery programme and either did (as comparators) or did not follow through with the surgery.

### Intervention(s) and exposure(s)

Bariatric surgical procedures as a result of obesity will be included that are classified as either restrictive, malabsorptive or both. These will include adjustable gastric banding, Roux-en-Y gastric bypass, sleeve gastrectomy, standard biliopancreatic diversion (Scopinaro), duodenal switch diversion, gastric plication, mini gastric bypass, vertical banded gastroplasty, loop gastric bypass, gastric balloon.

### Comparator(s)/control

The comparators in this study are bariatric surgery clients who were enrolled in a bariatric surgery programme, who did not withdraw from the bariatric surgery programme in the preoperative period and who did receive bariatric surgery.

### Outcome(s)

#### Primary outcomes

The primary outcomes of interest are any sociodemographic, medical, cultural, psychological, patient-led factors affecting preoperative attrition. Additionally, any other factors that arise from the literature can be included under these categories if they are deemed to contribute to patient attrition. The sociodemographic characteristics of interest are those affecting population-based bariatric surgery access also identified by Bhogal et al. [16] including place of residence, race/ethnicity, occupation, gender/sex, religion, education, language, socioeconomic status, and social capital.

Medical characteristics will include any medical issues associated with pre-operative attrition identified by a given study. Cultural aspects will include specific relational or social customs and traditions around health, the body, medicine, and food specific to a particular culture identified as contributing to preoperative bariatric surgery withdrawal. Psychological issues will include any mental illness such as depression, anxiety, schizophrenia, bipolar, and personality disorders. Patient-led factors will include client disengagement, voluntary withdrawal, impatience, level of knowledge, and attitudes and behaviours.

### Study characteristics (design, country of conduct, total number of subjects)

Inclusion: retrospective cohort, prospective cohort, case control and cross sectional studies.

Report characteristics: 1997–2020.

Language: articles written in English language only due to resource restrictions. Any non-English but potentially relevant studies will be listed in an ‘awaiting assessment’ section reserved for known studies that require investigation if the review is updated.

Publication status: published and unpublished

Number of subjects: the number who are enrolled into the bariatric surgery programme as per study retrieved.

### Search strategy

Two experienced researchers will conduct the literature search which will be peer-reviewed by two professors independent of the research team. The following electronic databases will be used to identify studies according to the predefined criteria: Scopus 1788–present, CINAHL 1980–present, PsycINFO 1806–present, Web of Science 1900–present, and MEDLINE 1996–present. A lower limit of articles published in the period from 1997 has been set because the surgery procedures used after this period have been shown to be robust with low surgical risks and significant results in comparison to earlier decades [3]. Reference lists of reviews and meta-analyses will be searched (but not included) to identify any relevant articles. Hand searches of references of selected studies will be conducted. An additional file gives search terms and strategy example for at least one electronic database (see Additional file 2).

The following websites will be searched for grey literature:

- Ministry of Health, New Zealand: http://www.health.govt.nz/
- Australian Government, National Health and Medical Research Council: https://www.nhmrc.gov.au/
- Statistics Canada: http://www.statcan.gc.ca/start-debut-eng.html
- National Institute for Health and Care Excellence: https://www.nice.org.uk/guidance
- National Centre for Health Statistics: http://www.cdc.gov/nchs/ www.cdc.gov/obesity/data/index.html
- Royal College of Physicians and Surgeons of Canada: http://www.royalcollege.ca/rcsite/home-e
- National Institutes of Health: http://www.nih.gov/
- Royal College of Surgeons of England: http://www.rcseng.ac.uk/
- Health and Social Care Information Centre: http://www.hscic.gov.uk/
- European Institute for Health Records: http://www.eurorec.org/
- Royal Australasian College of Surgeons: http://www.surgeons.org/
- Australian Institute for Health and Welfare: http://www.aihw.gov.au/
- U.S. Department of Health and Human Services, Agency for Healthcare Research and Quality: https://www.ahrq.gov/research/findings/factsheets/errors-safety/ngc/national-guideline-clearinghouse.html

### Data extraction, (selection and coding)

Titles and/or abstracts of studies retrieved using the search strategy and those from the grey literature will be identified and assessed for eligibility by two researchers (TT and NT) independently of each other to identify studies that potentially meet the inclusion criteria outlined above. The full text of these potentially eligible studies will then be assessed using full-text criteria by the same two researchers. Disagreement of including any particular study will be discussed until inclusion/exclusion agreement is reached. An audit will be taken out on ten randomly selected articles by two separate research team members (RT and WW) who will assess the appropriateness of the studies for selection according to the criteria. TT and NT will then extract data independently, and discrepancies will be identified and resolved through discussion. Extracted information will include study setting, study population and participant demographics and baseline characteristics, details of the bariatric surgery type, study methodology, and attrition and retention rates. Information will be extracted from each included study on the number and percentage of enrolled clients who did/did not receive bariatric surgery per study. All factors the selected studies have examined in relation to attrition will be listed, and factors that were significant (*p* ≤ .05) will be clearly identified: patient sociodemographic characteristics (place of residence, race/ethnicity, occupation, gender/sex, religion, education, socioeconomic status, social capital, age, health insurance status) and patient risk factors (BMI, comorbidities, smoking status, mental health status, quality of life, physical activity level).

Cultural reasons will include social customs and traditions around health, the body, food specific to a particular culture identified as contributing to preoperative attrition. Psychological information will include conditions such as depression, anxiety, personality disorders, schizophrenia, and autism. Patient-led and preoperative reasons cited for attrition will be extracted including client disengagement, voluntary withdrawal, impatience, level of knowledge, and attitudes and behaviours. In addition, the extracted data will include any other factors that can be included in these broader categories if they contribute to patient attrition.

### Study records

All systematic review documents will be stored on the principal investigator’s university drive, and when documents need to be shared with other research team members, they will be shared using a password-protected online cloud storage database. Literature searches will be alphabetised onto Microsoft Excel sheets so that duplicates can be filtered out. Extracted data from final review articles will also be synthesised using Microsoft Excel tables with factors listed underneath predefined attrition categories under review. The two researchers involved with auditing the ten randomly selected articles for full-article assessment will not have access to the shared online cloud-database. They will be given hard copies of the articles with a criteria scoring list to ensure independence of the audit.

### Risk of bias (quality) assessment

Two reviewers (TT and NT) will independently assess the risk of bias (quality) of each selected study using the Newcastle-Ottawa Quality Assessment Scale (NOS) for case-control and cohort studies and the AXIS assessment tool for critical appraisal of cross-sectional studies [19]. The NOS encompasses a ranking system for three risk of bias categories: selection (of cases or cohort), comparability (of cases or cohort), and outcome (this refers to the assessment of outcomes for case studies and ascertainment of exposure for cohort studies). The AXIS tool for cross-sectional studies involves a critical appraisal of a study’s reliability, worth, and relevance. The appraisal tool employs 20 questions with ‘yes/no’ or ‘do not know/comment’ responses aimed at informing a study’s quality. A third reviewer will be used as an arbitrator if the there is disagreement. All selected studies will be included in the review; however, the quality of studies and confidence the reviewers have in the studies’ quality test results will be commented on in the review.

### Strategy for data synthesis

Characteristics of included studies will be tabulated including patient study reference, study type, setting/context, all factors examined across studies, and statistically significant (*p* ≤ .05) associations. The results tabulated refer to the factors identified by the studies that were both non-significant and significantly related to preoperative attrition. The results will report bivariate analyses unless multiple regression analysis is available that will be considered more accurate due to controlling for confounders. Further, if a given study reports several regression models, results from the model with the largest number of predictors will be included. Additionally, frequency effect size will be calculated as the number of studies with a finding divided by the total number of included studies will be presented to reflect the proportion of studies in which a particular factor has been investigated. A narrative summary of the results that discusses connections between study factors will be presented, and a flow diagram with results of the literature search will be presented indicating the number of studies screened, the number of studies excluded, and reasons for exclusion.

### Dissemination plans

Publication of the review in a scientific peer-reviewed journal and dissemination at all relevant conferences and any poster opportunities will be pursued.

## Additional files

Additional file 1: PRISMA-P 2015 Checklist. (DOCX 71 kb)

Additional file 2: Search Strategy Example for at least one electronic database PsychINFO – 1806 to present. (DOCX 38 kb)

## Abbreviations

PRISMA: Preferred Reporting Items for Systematic Reviews and Meta-Analyses
PRISMA-P: Preferred Reporting Items for Systematic review and Meta-Analysis Protocols
