# Supplementary material for: Factors associated with preoperative attrition in bariatric surgery: a protocol for a systematic review
Source: Syst Rev. 2018 Nov 28;7:212. doi: 10.1186/s13643-018-0855-x (PMC6262965; doi:10.1186/s13643-018-0855-x)
Supplement: Supplementary file 2 — Search Strategy Example for at least one electronic database PsychINFO – 1806 to present. (DOCX 38 kb) [file 13643_2018_855_MOESM2_ESM.docx]

**Search Strategy Example for at least one electronic database PsychINFO – 1806 to present:**

|  | **Search Terms process example in PsychINFO – 1806 to present** | Hits | Relevant articles from hits | Minus duplicates |
| --- | --- | --- | --- | --- |
| 1 | ("Bariatric bypass surgery" OR "Bariatric Surgery" OR "Gastric Bypass" OR "Band surgery" OR "Jejunoileal bypass" OR "Laparoscopic surgery" OR "Obesity surgery" OR "roux-en-Y" OR "Sleeve Gastrectomy" OR " sleeve gastrectomy" OR "adjustable gastric band" OR "vertical banded gastroplasty" OR "biliopancreatic diversion" OR "duodenal switch" OR "mini-gastric bypass" OR "loop bypass" OR "gastric placation" OR "gastric balloon" OR "Scopinaro procedure" ) [PsycINFo = .mp, where .mp=title, abstract, heading word, table of contents, key concepts, original title, tests & measures] | 1513 |  |  |
| 2 | (Nonattendance OR "Did not attend" OR "Did not complete" OR Attrition OR Disengagement OR Withdrawal OR "Self-removal" OR Drop-out OR Retention OR Completion).mp | 120972 |  |  |
|  | 1 AND 2 | 35 | 15 | 15 |
|  | 15 related to bariatric surgery, 3 specific to preoperative period, a lot of articles on surgery in general and training medical students |  |  |  |
| 3 | (Equity OR Disparity OR Inequity OR inequality OR Psych* OR Soci* OR "Individual factors" OR Economic OR Co-morbidities OR "Mental illness" OR geographic OR "place of residence" OR race OR culture OR occupation OR gender OR sex OR religion OR "education level" OR "socioeconomic status" OR "social capital" OR "health insurance status" OR ethnic* OR Pacific OR "Patient risk factors" OR comorbidities OR "smoking" OR "mental health" OR "quality of life").mp | 2424044 |  |  |
|  | 1 AND 2 AND 3 | 23 | 12 | 0 |
|  | 9 related to bariatric surgery, 2 specific to the preoperative period. Many articles on surgery in general. |  |  |  |
|  | Edited terms to be more specific to bariatric surgery and refined the third set of terms to remove terms that were more about general surgery such as 'patient risk factors' and 'Health insurance status'. Then added 'impatience' to broaden the reasons for attrition scope. |  |  |  |
| 4 | (Preoperative OR Bariatric OR "Gastric Bypass" OR "Obesity surgery" OR "roux-en-Y").mp | 4909 |  |  |
| 5 | (Nonattendance OR "Did not attend" OR "Did not complete" OR Attrition OR Disengagement OR Withdrawal OR "Self-removal" OR Drop-out OR Retention OR Completion).mp | 111851 |  |  |
|  | 4 AND 5 | 154 | 17 | 2 |
| 6 | (Equity OR Disparity OR Inequity OR inequality OR Psych* OR Soci* OR Economic OR Co-morbidities OR "Mental illness" OR geographic OR race OR culture OR occupation OR gender OR sex OR religion OR education OR "social capital" OR ethnic* OR Pacific OR comorbidities "quality of life" OR Impatience).mp | 2522921 |  |  |
|  | 4 AND 5 AND 6 | 47 | 10 | 0 |
|  | 8 related to Bariatric surgery, 2 specific to preoperative period |  |  |  |
|  |  |  | Total relevant hits | 17 |

**Search Terms example for PsychINFO:**

"Bariatric bypass surgery" OR "Bariatric Surgery" OR "Gastric Bypass" OR "Band surgery" OR "Jejunoileal bypass" OR "Laparoscopic surgery" OR "Obesity surgery" OR "roux-en-Y" OR "Sleeve Gastrectomy" OR " sleeve gastrectomy" OR "adjustable gastric band" OR "vertical banded gastroplasty" OR "biliopancreatic diversion" OR "duodenal switch" OR "mini-gastric bypass" OR "loop bypass" OR "gastric placation" OR "gastric balloon" OR "ScopiNaro procedure" .mp

AND Nonattendance OR "Did not attend" OR "Did not complete" OR Attrition OR Disengagement OR Withdrawal OR "Self-removal" OR Drop-out OR Retention OR Completion OR Equity OR Disparity OR Inequity OR inequality OR Psych* factors OR Soci* factors OR "Individual factors" OR Economic OR Co-morbidities OR "Mental illness" OR geographic OR "place of residence" OR race OR culture OR occupation OR gender OR sex OR religion OR "education level" OR "socioeconomic status" OR "social capital" OR "health insurance status" OR ethnic* OR Pacific OR "Patient risk factors" OR comorbidities OR "smoking status" OR "mental health status" OR "quality of life".mp

Second Search:

"Bariatric bypass surgery" OR "Bariatric Surgery" OR "Gastric Bypass" OR "Band surgery" OR "Jejunoileal bypass" OR "Laparoscopic surgery" OR "Obesity surgery" OR "roux-en-Y" OR "Sleeve Gastrectomy" OR " sleeve gastrectomy" OR "adjustable gastric band" OR "vertical banded gastroplasty" OR "biliopancreatic diversion" OR "duodenal switch" OR "mini-gastric bypass" OR "loop bypass" OR "gastric placation" OR "gastric balloon" OR "Scopinaro procedure"  .mp

AND Preop* OR Pre-surg* or “before surgery”.mp

AND Nonattendance OR Attrition OR "Self-removal" OR Drop-out OR Retention OR Completion OR “noncompletion”.mp

Filters:

Report characteristics: 1997 - 2017

Language: English.

Publication status: Peer-reviewed
